# Understanding structure, function, and mutations in the mitochondrial ATP synthase

**DOI:** 10.15698/mic2015.04.197

**Published:** 2015-03-24

**Authors:** Ting Xu, Vijayakanth Pagadala, David M. Mueller

**Affiliations:** 1 Department of Biochemistry and Molecular Biology, The Chicago Medical School, Rosalind Franklin University of Medicine and Science, 3333 Green Bay Road, North Chicago, IL 60064.; 2 Department of Chemical Biology and Medicinal Chemistry, Eshelman School of Pharmacy, University of North Carolina, Chapel Hill, NC.

**Keywords:** ATP synthase, F1 ATPase, F1Fo ATP synthase, mitochondrial diseases, petite mutations, uncoupling

## Abstract

The mitochondrial ATP synthase is a multimeric enzyme complex with an overall molecular weight of about 600,000 Da. The ATP synthase is a molecular motor composed of two separable parts: F_1_ and F_o_. The F_1_ portion contains the catalytic sites for ATP synthesis and protrudes into the mitochondrial matrix. F_o_ forms a proton turbine that is embedded in the inner membrane and connected to the rotor of F_1_. The flux of protons flowing down a potential gradient powers the rotation of the rotor driving the synthesis of ATP. Thus, the flow of protons though F_o_ is coupled to the synthesis of ATP. This review will discuss the structure/function relationship in the ATP synthase as determined by biochemical, crystallographic, and genetic studies. An emphasis will be placed on linking the structure/function relationship with understanding how disease causing mutations or putative single nucleotide polymorphisms (SNPs) in genes encoding the subunits of the ATP synthase, will affect the function of the enzyme and the health of the individual. The review will start by summarizing the current understanding of the subunit composition of the enzyme and the role of the subunits followed by a discussion on known mutations and their effect on the activity of the ATP synthase. The review will conclude with a summary of mutations in genes encoding subunits of the ATP synthase that are known to be responsible for human disease, and a brief discussion on SNPs.

## INTRODUCTION

Peter Mitchell first proposed that the proton potential was used to provide the energy needed for the synthesis of ATP. While the chemiosmotic hypothesis 1 was proposed in 1961, because of the complexities of the enzyme, we are just now understanding the molecular details of ATP synthesis. The ATP synthase is a large multi-subunit enzyme complex composed of up to 20 different subunits or polypeptides that are in some cases present in multiple copies. The ATP synthase is bound to the inner membrane of the mitochondrion with a number of subunits being water-soluble while others are integral membrane proteins.

The mitochondrial ATP synthase is composed of two separable components: F_1_ (factor 1) and F_o_ (factor that confers sensitivity to oligomycin). The ATP synthase is a reversible molecular motor comprised of two parts: a proton turbine (within F_o_) and a molecular machine (F¬_1_) that uses rotational energy to form ATP from ADP and phosphate. The proton turbine is powered by the flow of protons down a potential gradient across the mitochondrial membrane created by the electron transport chain during respiration. The rotor of the turbine is within the F_1_ and when it rotates, drives the synthesis of ATP in the general mechanism described by the binding change hypothesis of Paul Boyer 2.

The ATP synthase acts to convert the energy of oxidation-reduction reactions of the electron transport chain (respiration) to the phosphorylation of ADP. The synthesis of ATP is “coupled” to the respiratory chain via the proton potential. The number of ATP molecules made, per number of atoms of oxygen reduced in the electron transport chain, (P/O ratio) is a measure of the coupling of ATP synthase with the electron transport chain 3. The P/O ratio is dependent upon a number variables, including the membrane integrity, site of the electron transport chain where the electrons are accepted, and the integrity of the ATP synthase. Because of these variables and basic experimental variations, a range of P/O have been reported, but the consensus values for mammalian mitochondria, are 2.5 and 1.5, for electrons entering at NADH dehydrogenase and succinate dehydrogenase, respectively 4,5. The recent understandings of the structure/function relationship provide insight into the coupling capacity of the ATP synthase partly based upon the theoretical number of ATP molecules made per flux of protons. Using the c-ring stoichiometry of 8 (see discussion on F_o_ below) for the bovine enzyme, the maximal theoretical P/O ratio for mammalian mitochondria is 2.7 for NADH and 1.6 for succinate 3. Yeast *S. cerevisiae* lacks the first coupling site 6 and considering the yeast c-ring stoichiometry of 10, the P/O ratio for NADH and succinate is about 1.3. However, for analysis of the ATP synthase function, given that all variables outside the ATP synthase are equal, a higher P/O ratio corresponds to a higher efficiency of the enzyme. Classical chemical uncouplers, such as 2,4 dinitrophenol, allow respiration without ATP synthesis thus reducing the P/O ratio to zero, by dissipating the proton potential across the mitochondrial membrane. However, the flow of protons through F_o_ into the matrix is also coupled - coupled to the phosphorylation of ADP, and mutations can alter the coupling which reduces the P/O ratio.

## STRUCTURE AND ORGANIZATION OF THE F_1_F_o_ ATP SYNTHASE

**Figure 1:**
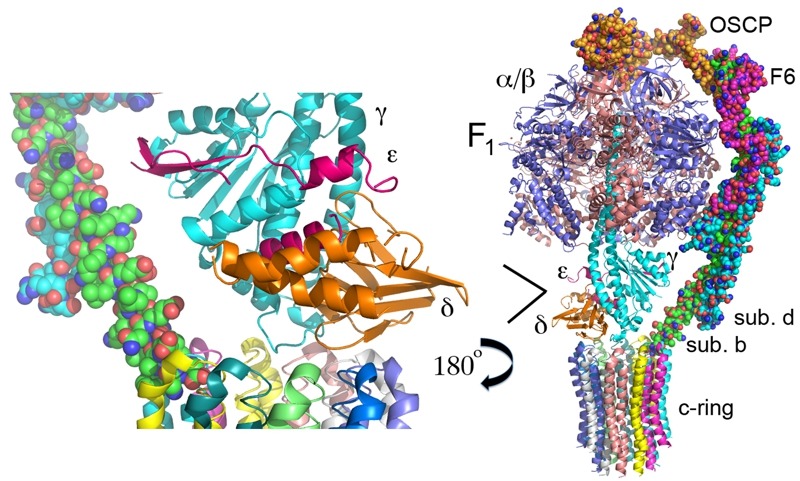
FIGURE 1: Structural representation of the ATP synthase. A composite representation of the ATP synthase is shown on the right with the F_1_, subunits of the stator, and the c_10_-ring shown. The structure of the F_1_ and c_10_-ring were derived from the x-ray crystal structures (2HDL and 3U2F) while the structures of the stator (peripheral stalk) were derived from the x-ray crystal structures of the bovine components (2WSS and 2CLY). Note, that a number of subunits have not been represented in the structure, most notably, subunit-a, which forms the proton half-channels.

A composite model of the mitochondrial ATP synthase is shown in Figure 1. This composite model was derived from the crystal structure of the yeast F_1_ ATPase (pdb: 2HLD, the yeast c_10_-ring, 3U2F, and the bovine stator derived from two structures, 2WSS and 2CLY 7,8,9,10,11). Table 1 lists the subunit composition of the bovine, yeast and *E. coli* enzymes along with the genes and relevant information on the structure/function relationship. The composite model is missing components of F_o_, which provide access for protons from the matrix and intermembrane space to the proton acceptor/donor site of the c-ring, which is positioned in the center of the lipid bilayer of the inner mitochondrial membrane. Based on subunit composition, the bacterial enzyme is much less complex than the mitochondrial enzyme, which may be due to the greater need for regulation in the eukaryotic cell. However, while the details differ, the general mechanism of ATP synthesis is conserved from the bacterial to the mammalian enzyme. The structure/function relationship of the subunits will be discussed in three groups: F_1_, F_o_, and stator.

**Table 1:** f: N-formyl Met, ac: putative acylation, m: mature; +The N-terminus of the mature subunit was derived by many investigators, but a more comprehensive list by Arnold et al., 71.

Mammalian	alias	*E. coli*	Yeast	#	Residues+	MW kD	Yeast Gene	Human Gene	Genome	Role	Sector	Importance
α	sub. 1	α	α	3	510m	54.9	ATP1	ATP5A1	nuclear	catalytic	F_1_	Essential
β	sub. 2	β	β	3	478m	51.1	ATP2	ATP5B	nuclear	catalytic	F_1_	Essential
γ	sub. 3	γ	γ	1	278m	30.6	ATP3	ATP5C1	nuclear	rotor	F_1_	Essential
δ		ε	δ	1	138m	14.6	ATP16, ATPδ	ATP5D	nuclear	rotor	F_1_	Essential
ε		none	ε	1	61m	6.1	ATP15, ATPε	ATP5E	nuclear	rotor	F_1_	Important
OSCP	sub. 5	δ	OSCP	1	195m	20.9	ATP5	ATP5O	nuclear	stator		Essential
sub. a	sub. 6	sub. a	sub. a	1	259	29.1	ATP6	ATP6	mito	proton pore	F_o_	Essential
sub. b	sub. 4	sub. b	sub. b	1	209m	23.3	ATP4	ATP5F1	nuclear	stator	F_o_	Essential
sub. c	sub. 9	sub. c	sub. c	10	f76	7.76	ATP9	ATP5G1, ATP5G2, ATP5G3	nuclear (mammalian) /mito (yeast)	proton pore	F_o_	Essential
sub. 8	A6L	none	sub. 8	1	f48	5.8	ATP8	ATP8	mito	stator	F_o_	Essential
sub. d	sub. 7	none	sub. d	1	173m	19.7	ATP7	ATP5H	nuclear	stator	F_o_	Essential
sub. e	TIM11	none	sub. e	1	ac95	10.7	ATP21/TIM11	ATP5I	nuclear	stator	F_o_	Dispensable
sub. f		none	sub. f	1	95m	10.6	ATP17	ATP5J2	nuclear	stator	F_o_	Essential
sub. g		none	sub. g	1	115m	12.9	ATP20	ATP5L, ATP5L2	nuclear	stator	F_o_	Dispensable
?	sub. j	none	sub. i	1	59m	6.7	ATP18	?	nuclear	??		Important
?		none	sub. k	1	68m	7.5	ATP19	?	nuclear	stator		Dispensable
F6		none	sub. h	1	92m	10.4	ATP14	ATP5J	nuclear	stator		Essential
IF1		none	Inh1p	1	63m	7.4	INH1	ATPIF1	nuclear	regulatory		Dispensable
?		none	Stf1p	1	63m	7.3	STF1	?	nuclear	regulatory		Dispensable
?		none	Stf2p	1	84	9.6	STF2	?	nuclear	regulatory		Dispensable

### The F_1_ ATPase

The mitochondrial F_1_ ATPase has the subunit composition α_3_β_3_γδε. The molecular weight of the subunits are 55 kDa, 51 kDa, 30 kDa, 15 kDa, and 5.7 kDa for the mature bovine α, β, γ, δ, and ε subunits, respectively. The X-ray crystal structure of the bovine F_1_-ATPase provided the first look at the structure/function relationship of the subunits in the F_1_ ATPase 12 and subsequent structures, some with bound inhibitors, have given greater understanding into the molecular mechanism of ATP synthesis 13,14,15,16,17,18,19,20,21,22,23. The α- and β-subunits share only about 20% identity but they form nearly identical folds and the α-carbon atom positions differ only by about 2.8 Å rmsd (root mean square deviation) with the greatest spatial divergence in the last 40 residues. The nucleotide binding sites are at the interfaces between the α- and β-subunits with the catalytic site formed primarily by the β-subunit and the non-catalytic site formed primarily by the α-subunit. In accordance, there are 6 nucleotide-binding sites in the α_3_β_3_γ assembly with 3 being catalytic binding sites and 3 being non-catalytic binding sites. Support for the residues involved in the binding and catalysis deduced from the crystal structure was obtained by extensive mutagenesis of primarily the *E. coli* enzyme, prior to and after the structure determination (for review, see 24). The P-loop (residues 156-163) is integral to the catalytic site with β-subunit residues Gly161/Lys162/Thr163 being nearly invariable with only Ser163 being a functional variant 25. For ATP hydrolysis, β-Glu188 has been concluded to act as a catalytic base that activates a water molecule in a reaction mechanism that has been described as S_N_2 26 where in the transition state, the γ-phosphate assumes a trigonal-bipyramidal arrangement of the oxygen atoms. ATP hydrolysis is the reverse of ATP synthesis and microscopic reversibility is widely accepted for this reaction. β-Asp256 and the β- and γ-phosphates for ATP form the Mg^2+^ binding site. Arg373 of the α-subunit is essential for catalysis and the crystal structure suggests that it is involved in stabilizing the penta-coordinate transition state. This role was more clearly demonstrated in the structure of the bovine F_1_ ATPase with the transition state analogue, ADP:AlF_4_, which showed the guanidinium group of α-Arg373 in a position to stabilize the transition state 18,27. In the structure of the yeast F_1_ ATPase, α-Arg373 is also shown to participate in the binding of inorganic phosphate, one of the substrates for the synthesis of ATP 7.

The crystal structure of the F_1_ gives clues as to why the non-catalytic sites are catalytically inactive. The relative position of the nucleotide bound in the catalytic and non-catalytic sites are nearly identical suggesting that the nucleotide-binding site is conserved. In the non-catalytic site, β-Arg356 is replaced by α-Arg373, α-Asp269 replaces β-Asp256, and α-Gly174/α-Lys175/α-Thr176 are replaced by β-Gly161/β-Lys162/β-Thr163. However, β-Glu188 is replaced by α-Gln208 thereby removing the carboxylate that would otherwise act as a catalytic base. In addition, as will be discussed below, the structure of the catalytic sites alternate between a closed and open conformation during the catalytic cycle but all three of the non-catalytic sites are in the closed conformation. The absence of a catalytic base and the inability to transition between different catalytic conformations, as evidenced by the absence of open conformation, are major determinants in the catalytic incapacity of the non-catalytic sites.

The seminal x-ray structure of the bovine F_1_ ATPase supported a rotational mechanism for ATP synthesis 12. The three catalytic sites were different in both structure and occupancy; sites were occupied with AMP-PNP, ADP, and the third catalytic site had no bound nucleotide (empty). As such, the active sites were named, TP, DP, and E and the corresponding β-subunits were named, β_TP_, β_DP_ and β_E_. The structure of the yeast F_1_ ATPase showed very similar results but the DP site was occupied with AMP-PNP 7. The structure of the nucleotide-binding site in E was open and largely different from either DP or TP and this explained why no nucleotide was bound to β_E_. For instance, α-Arg373 is displaced 7.2 Å in the E site as compared to the TP or DP site and is not in position to interact with bound nucleotide. In the structure of the yeast F_1_ ATPase, inorganic phosphate (or possibly sulfate) was bound in β_E_ supporting the conclusion that β_E_ is a catalytically important state 7. The asymmetry of the catalytic sites is determined by the position of the γ-subunit, which is located in the center of the α_3_β_3_ core. The structure suggested that the conformation of the catalytic site is determined by the position of the γ-subunit, which rotates relative to the α_3_β_3_ core in 120^o^ increments during the catalytic cycle. Indeed, this was later proven by direct visualization of the movement of actin filaments fixed to the γ-subunit of bacterial F_1_ ATPase due to hydrolysis and subsequently by demonstration of ATP synthesis driven by physical rotation of the rotor 28,29,30. This mechanism was also consistent with the binding change site hypothesis for ATP synthesis proposed by Boyer 2. Thus, the γ-subunit is a rotor that rotates within the α_3_β_3_ catalytic core effecting conformational changes in the nucleotide binding sites thereby effecting ATP synthesis.

**Figure 2:**
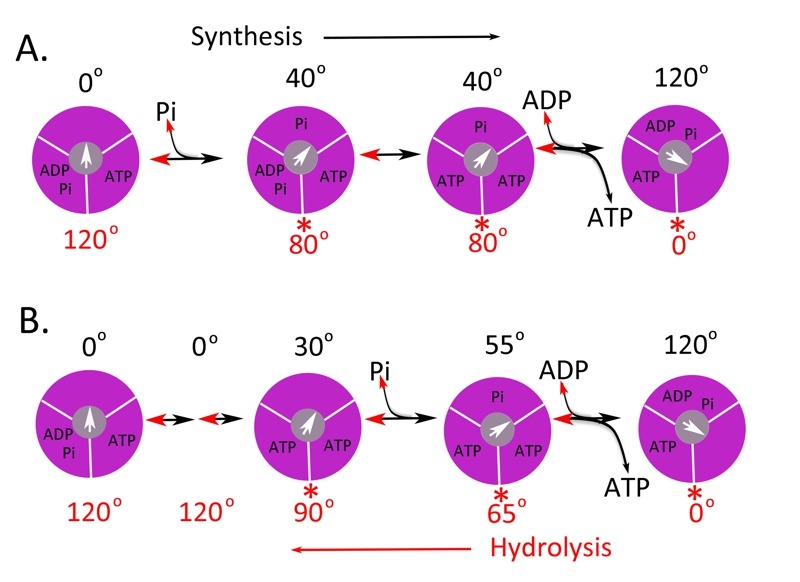
FIGURE 2: Possible scheme for site occupancy during ATP synthesis. Shown is the one possible mechanism for ATP synthesis based on the data for the bacterial 34 and yeast 35 enzyme (A) and the human (B) F_1_ ATPase 41. The direction from left to right is for ATP synthesis and right to left for ATP hydrolysis. The three binding sites are shown in magenta and the γ-subunit in grey with the arrow indicating the relative position. The asterisks (*) indicate the dwell angle observed during ATP hydrolysis.

The molecular details of the motor movement of the synthetic pathway for ATP synthesis have been largely studied by determining the hydrolytic pathway with the assumption of microscopic reversibility. Single molecule experiments have mostly been done using the F_1_ from *Bacillus* *PS3* (28,31,32,33,34,35,36,37,38,39,40) however, experiments using enzyme from *E. coli*, yeast, and human have also been reported 35,37,38,39,40,41. The results using F_1_ from *Bacillus*, *E. coli*, and yeast generally agree, but the details of the mechanism differ slightly from that of the human enzyme. Figure 2 shows a molecular mechanism for the synthesis of ATP based on the studies for the hydrolysis of ATP by the bacterial and human enzymes 41 and also based on the structure of the bovine enzyme 12. For the bacterial and yeast enzymes, there are 2 dwell positions during ATP hydrolysis, which can vary slightly: one at 0^o^, which corresponds to ATP binding and one at 80-90^o^ during the catalytic reaction. For the *Bacillus PS3 *enzyme, Pi release also occurs at 80^o^. For the human enzyme, there are 3 dwell positions during ATP hydrolysis: at 0^o^ due to ATP binding, at 65^o^ due to Pi release, and at 90^o^ due to ATP hydrolysis. The steps occur 3 times for a full rotation and thus each step is repeated in 120^o^ increments.

In terms of ATP synthesis for the human enzyme, at 0^o^, F_1_ is proposed to contain ADP and Pi in one site and ATP in a second site, while the third site is empty. The γ-subunit rotates by 30^o^, which is followed by the phosphorylation of ADP to ATP. Pi binds to the empty subunit followed by the rotation of the γ-subunit by 25^o^. The final step consists of the binding of ADP, the rotation the γ-subunit by 65^o^ and the release of ATP made in a prior round. In this scheme, the newly bound ADP and Pi are not converted to ATP until the second round, and the newly formed ATP is not released until the third round. This mechanism fits nicely with the biochemical and structural properties of the enzyme. The three sites identified by crystallography of the bovine enzyme 12, TP, DP, and E sites, relate to the ground state structure likely at about 65^o^ in this scheme. The conversion of ADP and Pi to ATP has been shown to be isoenergetic by both O^18^ exchange studies and hydrolysis of ATP under unisite conditions 2,42,43,44,45. During ATP hydrolysis, there are 2 steps that provide torque: the release of Pi and ATP binding 31,34,46. In the synthesis direction, energy would be needed for the binding of Pi and the release of ATP. In this scheme, all three sites are cooperating in the synthesis of ATP; one site is making ATP, the second site is binding ADP and Pi, and the third site is releasing ATP.

This mechanistic scheme predicts that the inhibition of one of the three catalytic sites will completely inhibit the enzyme. To assess this, the *Bacilus PS3* enzyme was prepared, containing 1, 2 and 3 β-subunits with the catalytic base mutation, β-E190Q 47. ATPase activity of the enzyme with just one of three β-catalytic sites mutated was completely inhibited confirming that all three catalytic sites must be functional for activity. A similar study was done using mutations (“slow”) in the β-subunit that reduced the binding constant for ATP to the catalytic site 48. In this case, slowing the binding of ATP at one site had no effect on the rate of ATP binding at the other 2 sites. At a molecular level, the β-E190Q mutation acted in a dominant manner, while the “slow” mutations acted in a co-dominant manner. These results have implications in understanding the genetics of disease causing mutations in the α- and β-subunits of the ATP synthase.

The rotation of the γ-subunit within the core of the F_1_ ATPase causes the conversion of the conformation of the active site from E to DP to TP. There are 2 primary sites of interaction between the β-subunit, which largely forms the active site, and the γ-subunit, which forms the rotor and these regions are referred to as Catch 1 and Catch 2. Catch 1 includes residues in a loop formed by residues 312-319 in the β-subunit and residues in a helix of the γ-subunit formed by residues 252-255. Catch 2 encompasses residues within a loop from 387-398 in the β-subunit and residues 87-90 and 235-242 in the γ-subunits 12.

The rotor, also called the central stalk, is composed of the γ-subunit along with the δ- and ε-subunits. The structures of the δ- and ε-subunits were not entirely visible in the first structure of the bovine F_1_ ATPase but became clear in the structure of the bovine F_1_ ATPase inhibited with *N,N’* dicyclohexylcarbodiimide (DCCD) 17. The belief is that the inhibition by DCCD was incidental to the resolution of the δ- and ε-subunits and not causative. The δ- and ε-subunits are positioned at the base of the rotor and appear to sit close to the F_o_ portion of the ATP synthase. As is discussed below, mutagenesis experiments suggest that the γ-, δ- and ε-subunits share a common function in enzyme coupling - i.e., coupling proton translocation though F_o_ with rotation of the rotor.

### Factor F_o_

Factor F_o_ is also commonly referred to as F_0_ (F zero). Efraim Racker named factor F_o_ for the factor that conferred sensitivity of the F_1_ ATPase to the antibiotic, oligomycin 49,50,51. Oligomycin is now known to bind to subunit-c of F_o_ and prevent proton flow. Recently, the structure of oligomycin bound to yeast subunit c has been determined by X-ray crystallography at 1.9 Å resolution providing molecular details into oligomycin binding and an understanding of how oligomycin blocks proton translocation 52.

Based on the structural and genetic data, it is postulated that oligomycin binds on the face of the c-ring at the region comprised of the proton half channel, formed in part by subunit-a 52. The F_o_ sector is defined by the number of subunits in the biochemical preparation, all of which have the common characteristic of being membrane proteins or tightly associated with the membrane proteins. The F_o_ portion of the ATP synthase comprises the proton turbine as well as the base of the stator, while the rotor is composed of γδε 53. The core of F_o_ is composed of an oligomer of the c-subunits, which contain an essential carboxylate from the side chain of either a glutamate or aspartate residue. The side chain carboxyl acts as the proton donor and acceptor in proton translocation pathway. Two half-channels (or pathways) are postulated to exist and are thought to be formed by subunits-a, which provide access to the essential carboxylate on subunit-c and to the aqueous phase of the matrix and intermembrane spaces of the mitochondrion 54,55,56. Other subunits, such as A6L, may either modulate or provide structural support for the F_o_ portion of the enzyme. A recent model of the bovine ATP synthase using cryo-electron microscopy was able to show the arrangement of subunits a, b, c, e, f, g, and A6L, within the membrane domain 57.

The number of c-subunits varies between species with known stoichiometries ranging from 8-15 3,58,59,60,61,62. Bovine F_o_ is composed of a c_8_-ring 3 while yeast F_o_ is composed of a c_10_-ring 8,58. The c-ring rotates within the membrane in steps determined by the number of c-subunits in the c-ring. As such, movement of 8 protons are used to rotate the bovine c-ring 360^o^ driving the synthesis of 3 ATPs. This analysis provides insight into the thermodynamic considerations for ATP synthesis and provides theoretical values for ATP/H^+^ ratios and therefore, P/O ratios 3.

The essential glutamate is located in the center of helix 2 of the c-ring subunit, which positions it in the middle of the lipid bilayer. Thermodynamic considerations suggest that only the protonated form of glutamate is able to exist in the center of the bilayer and protonation/deprotonation reaction must occur in a hydrophilic environment created by two half-channels. The half-channels provide access to either the matrix or the intermembrane space thereby allowing proton flow between the compartments. The half-channels are thought to be comprised of subunit-a, but may also include subunit b and other components of F_o_. There is only one strictly conserved residue in subunit-a, Arg210 (*E. coli* numbering, Arg259, human), which is believed to be a principle component in the proton translocation pathway (see 63 for review). Recent electron microscopy images of the *Thermus thermophilis* V-ATPase and the bovine ATP synthase suggest that the contact between F_o_ and the half-channel is surprisingly small 57,64. This loose association may be essential for the high-speed rotation of the c-ring.

### The stator

The stator is also referred to as the peripheral or extrinsic stalk. The stator functions to hold F_1_ fixed to allow rotation of the rotor within the core of F_1_. The stator provides a structural support and is not involved directly in the catalytic reaction. The stator is composed of the oligomycin sensitive conferring protein (OSCP, subunit 5), subunit b, subunit d, and F6 and the X-ray structure has been solved for the peripheral stalk of bovine enzyme 9,11. Subunit b is anchored to the membrane while OSCP is locked to the top of F_1_. (The naming of subunit 5 as OSCP is unfortunate since OSCP does not participate in forming the oligomycin-binding site.) OSCP was so named because oligomycin prevents proton flow through F_o_ and inhibits ATP hydrolysis only if F_1_ is functionally attached and coupled to proton movement. Breaking the stator uncouples ATP hydrolysis from proton translocation because the F_1_ core can spin instead of the rotor. The primary structure of the stator proteins shows low conservation between bovine and yeast, as evidenced by yeast subunit h and bovine F6, which have just 14.5% sequence identity. However, the function is conserved because expression of a cDNA encoding bovine F6 complements the deletion mutation of the gene encoding subunit h in yeast 65.

### Other subunits

Subunit f is a component of the bovine and yeast ATP synthase 53,66,67, however, the role is uncertain. Subunit f associates with F_o_ and deletion of the gene in yeast results in phenotypes that are typical for an ATP synthase that is uncoupled 67 (see below). The role of subunit f is still ambiguous as loss of either a component of the peripheral stalk or F_o_ give similar phenotypes. Yeast with a null mutation in the corresponding gene is unable to grow on a nonfermentable carbon source and loses mitochondrial DNA at a high rate 67. As such, subunit f is an essential component of the mitochondrial ATP synthase.

Subunit i (aka subunit j) was identified as a component of the yeast ATP synthase nearly coincidently by two laboratories, which is why it has an alias 68,69. A mammalian homologue for subunit i has not been identified though this does not exclude its presence. Subunit i easily dissociates from the ATP synthase complex, which explains why it had not been identified in prior studies. Deletion of the gene encoding subunit i gave different results in the two laboratories. In one case, deletion of the gene encoding yeast subunit i does not alter the assembly of the ATP synthase; the resulting ATP synthase is functional, but is less well coupled based on the P/O ratio and growth phenotype of the yeast 68. However, in the second laboratory, deletion of the gene encoding subunit i resulted in complete loss of the ATP synthase with absence of the mitochondrial encoded subunits 69. These results are not as different as they appear because, a loss of coupling of the ATP synthase will result in the loss of mitochondrial DNA 70. As such, these results differ by a matter of degree, which can be caused by different growth conditions or genetic background of the yeast strains. While the role of subunit i is uncertain, it does appear to provide a role in the efficient coupling of the yeast ATP synthase.

A dimer form of the ATP synthase has been shown in yeast 71 and there is evidence of dimer forms of the ATP synthase in bovine, chloroplast, and other species of fungi 72,73. The dimer form appears to be involved in formation of cristae of the inner mitochondrial membrane, which may be why the dimer form has not been shown for the bacterial enzyme 74,75. Subunits e, k, and g are selectively associated but only subunits e and g are necessary for dimer formation 71,75. Subunit k has not been identified in the mammalian ATP synthase although this does not exclude the possibility of a mammalian homolog. Deletion of the gene encoding subunits e or g has pleiotropic effects including a decrease of cytochrome oxidase activity 76,77. While it is not uncommon for mutations in genes encoding mitochondrial proteins to exhibit pleiotropic effects, this effect was unusual in that the levels of cytochromes a + a_3_, c + c_1_, and b were unaltered suggesting that activity could be altered without altering synthesis or stability of the cytochromes. This and an unrelated study 78 suggest a link, which will be discussed later, between the ATP synthase and the respiratory chain.

The natural inhibitor protein, IF1, is thought to regulate the ATPase activity of the ATP synthase 79. The inhibitor protein has a unique characteristic in that it inhibits ATP hydrolysis, but not ATP synthesis. In yeast, there are also related molecules, Stf1p and Stf2p. Stf1p appears to stabilize the interaction of IF1 with F_1_ or possibly is an isoform of IF1 80. Deletion of the gene encoding IF1 from yeast results in mitochondria that demonstrate uncontrolled hydrolysis of ATP in the presence of a chemical uncoupler, such as 2,4 dinitrophenol 81. A number of studies have suggested that inhibitor protein has a role in preventing hydrolysis of ATP under ischemic conditions in mammalian heart 82,83,84,85,86 and other metabolic effects 87. While this is a conceptually attractive hypothesis, the energy of the hydrolysis of ATP by the ATP synthase is not lost - it is coupled to the pumping of protons across the mitochondrial membrane creating a proton gradient. In contrast, the hydrolysis of ATP by free F_1_ ATPase is futile and wasteful, and it would be important for the cell to control this hydrolysis. Free F_1_ ATPase has been suggested to be an intermediate in the assembly pathway of the ATP synthase 88. Thus, IF1 may serve a role in preventing the hydrolysis of ATP by free F_1_ that occurs during biogenesis of the ATP synthase complex. Stf2p appears to be important in yeast during times of stress or dehydration 89. Interestingly, over-expression of Stf2p in yeast results in a reduction of reactive oxygen species (ROS) in response to stress and thus provides a possible target to reduce ROS production in humans. In this regard, IF1 has been shown to be important for the survival of HeLa cells when they are exposed to high concentrations of ROS, but IF1 is not important for cell survival under normal conditions 90.

The X-ray crystal structure of the bovine inhibitor protein bound to the bovine F_1_ ATPase has shown that IF1 is trapped between the pair of the α- and β-subunits, which form the DP site 91,92. The mechanism of inhibition of IF1 was described in terms of the binding change hypothesis, but the explanation will be given in terms of the nomenclature used for the active sites of the ATP synthase. IF1 is postulated to initially bind to the open site (E), which closes to form the TP:IF1 site during ATP hydrolysis. A second round of ATP binding and hydrolysis converts the TP:IF1 site to the DP:IF1 site where it is locked in the final inhibited form. IF1 is thought to prevent the conversion of the DP:IF1 site to the E:IF1 site by steric interference within the interface of the α- and β-subunits. The reason as to why IF1 is unable to inhibit the reverse reaction, ATP synthesis, is still uncertain. The structural studies 91 do not support its role as a ratchet, which has been proposed for the inhibitory mechanism of the bacterial ε-subunit 93. The trivial explanation is that the conditions, such as pH and ΔΨ, during ATP synthase differ from conditions under which ATP hydrolysis occur, and IF1 does not bind to F_1_ under synthesis conditions.

### Deletion of genes encoding F_1_ subunits in yeast

A mutation that completely eliminates expression of a gene - a null mutation - is the simplest mutation to understand both at the level of the structure/function relationship of the protein and in relation to clinical relevance of the mutation. However, there are at least 2 instances with added clinical relevance and understanding of structure/function of the protein of the null mutation. First, haploinsufficiency is the situation where loss of one of the wild type alleles in a diploid organism causes a disease. Thus, a 50% drop in expression level is sufficient to cause disease or defective phenotype. Second is the situation where a null mutation causes a dominant negative or gain of (dysfunctional) activity. Both of these cases appear to be applicable in the yeast model system of null mutations in genes encoding subunits of the ATP synthase as well as for the yeast vacuolar ATPase 94,95.

All five subunits of the mitochondrial F_1_ ATPase are essential for the function of the ATP synthase. A priori, it would be predicted that deletion of any one of the five genes encoding the subunits of F_1_ would result in similar, if not identical, phenotypes. However, the situation is much more complicated than predicted 70. The phenotypes of the yeast strain with a deletion mutation in either the gene encoding α or β, were typical and not surprising; the cells were defective in oxidative phosphorylation and did not grow on medium with a nonfermentable carbon source. Different from the phenotypes caused by loss of α or β, yeast cells with a deletion of the gene encoding γ result in very small colonies and the cells had completely lost their intact mitochondrial DNA, i.e., cytoplasmic petite (ρ^-^/ρ^0^). However, deletion of genes encoding α and γ (ΔαΔγ) in the same cell results in cells with a phenotype that is similar to that of yeast with a deletion mutation just in the gene encoding α (Δα). Similarly, cells with a deletion mutation in the gene encoding β had the same phenotype as cells with double mutation, ΔβΔγ. Deletion of the gene encoding δ from yeast resulted in a phenotype that was similar to those obtained with deletion of the gene encoding γ, but not as dramatic. Similar to cells that have a deletion in the gene encoding the γ-subunit, cells with a deletion of the gene encoding δ caused the cells to lose 100% of their intact mitochondrial DNA. Lastly, deletion of the gene encoding ε had the weakest effect on the phenotype, but 60% of the cells became cytoplasmically petite. In each case, the percentage of cells that became cytoplasmically petite returned to normal level when a null mutation in the gene encoding either α or β was added. This effect indicates that the phenotype caused by the deletion of the gene encoding either α or β, was epistatic to that caused by deletion of the gene encoding either the γ, δ or ε-subunit.

Mutations in genes encoding chaperones, ATP11 or ATP12, had the same epistatic effect, in respect to mutations in the genes encoding the γ-, δ-, and ε-subunits, as deletion mutations in the genes encoding α or β. Atp11p and Atp12p are needed for the assembly of the α- and β-subunits into F_1 _and yeast strains with mutations in either ATP11 or ATP12 have phenotypes similar to yeast strains with mutations in the genes encoding the α and β-subunits 96,97,98,99,100,101. A reasonable explanation for the effect was that a subcomplex of the ATP synthase could assemble without one of the rotor subunits, but not without α or β, and the subcomplexes devoid of a rotor subunit were responsible for the additional phenotypes.

The effect by the loss of a subunit in the rotor can be deduced after considering the role of the rotor. With loss of the rotor, one would predict that the F_1_F_o_ subcomplex would allow protons to flow through F_o_ without F_o_ being coupled to the rotation of the rotor. Under this condition, the enzyme would be completely uncoupled and the mitochondria would exhibit a P/O ratio of zero. However, cells lacking γ or δ become 100% cytoplasmically petite. Since F_o_ subunits are encoded in the mitochondrial DNA, it is impossible to assess defects in coupling when the strain becomes entirely petite. Two different approaches helped resolve this problem. First, diploid yeast cells that were heterozygote for a mutation in the gene encoding γ or δ were impaired, but not totally defective in oxidative phosphorylation. Analysis of mitochondria isolated from these strains indicated that the deletion mutations caused an uncoupling of the ATP synthase presumably due to a leakage of protons into the mitochondria via the uncoupled ATP synthase subcomplex 102. The second approach used a doxycycline-regulated expression system to gradually reduce the level of δ in the cell 103. Using this system, direct evidence was obtained that the ATP synthase could assemble in the absence of δ and that this δ-less ATP synthase subcomplex was uncoupled because of a F_o_ mediated proton leak.

A central question that was left unanswered in this study was the relationship between uncoupling and loss of mitochondrial DNA, i.e. cytoplasmic petite formation. While this has not been conclusively answered, there is a reasonable explanation that is consistent with what is known about mitochondrion biogenesis 104. The biogenesis of the mitochondrion is dependent on the import and processing of proteins that are encoded in the nucleus and made in the cytoplasm. The import of the mitochondrial precursor proteins from the cytoplasm into the mitochondrion is dependent on proton potential across the mitochondrial membrane 105,106. (In petite strains or under anaerobic conditions, the mitochondrial membrane potential is generated by the electrogenic exchange of cytoplasmic ATP^4-^ with matrix ADP^3^ 107,108,109.) As such, uncoupling of the mitochondrial membrane will prevent the import of newly synthesized proteins and thus inhibit mitochondrion biogenesis. While yeast is a facultative anaerobe and able to survive without oxidative phosphorylation, all eukaryotic cells require mitochondrion and thus inhibiting the biogenesis is lethal. If the ATP synthase is uncoupled, the cell can only survive if the proton leak is blocked and that is most easily achieved in yeast by eliminating the mitochondrial DNA, which encodes subunits a, c and 8 which comprise the proton pathway. Thus, there is a correlation between the degree of uncoupling of the ATP synthase and the propensity of the cells to become cytoplasmic petite.

The cellular phenotypes and biochemical consequences associated with deletion of genes encoding α, β, γ, δ, and ε, provided key insights into role of the δ and ε subunits in the coupling of the enzyme. The deletion mutations in the genes encoding the γ, δ, or ε subunits resulted in an enzyme that was uncoupled and as such, provides a basis for recognizing missense mutations that uncouple the ATP synthase. Even the loss of a single copy of the genes encoding the γ or δ-subunits in a diploid strain can result in a dysfunctional uncoupling activity, i.e., haploinsufficiency 102.

The mitochondrial ε-subunit is unique in that it is the only F_1_ subunit for which there is no bacterial homolog. As with deletion of the gene encoding subunit i, deletion of the yeast gene encoding ε has given different phenotypes depending on the laboratories; in one case the loss of ε inactivated the enzyme while in the other case, its loss severely compromised the enzyme based on the growth phenotype on a non-fermentable carbon source 110,111. This discrepancy has been apparently resolved by the identification of selective mutations in the genes encoding either subunit a and c, which suppressed the growth phenotype in the yeast strain with the null mutation in the gene encoding the ε-subunit 112. It was suggested that the strains with a null mutation in the gene encoding the ε-subunit that were able to grow on a nonfermentable carbon source, had picked up a suppressor mutation in either the gene encoding subunit a or c.

### Missense mutations that uncouple the ATP synthase

The coupling of the ATP synthase is defined here as the coupling of ATP synthesis or hydrolysis with the movement or pumping of protons. Mutations that inhibit activity of the ATP synthase can act either by inhibiting the enzymatic reaction, as in an active site mutation, or by uncoupling proton movement with the reaction cycle. The coupling of rotation of the rotor with flow of proton and the synthesis of ATP is an intricate mechanism caused by numerous inter-subunit contacts. As such, mutations exist that alter these interactions and affect coupling. A genetic selection scheme, seemingly unrelated to the ATP synthase, selected cells with mutations in genes encoding the ATP synthase and these mutations cause an uncoupling of the ATP synthase.

Cytoplasmic “petite” yeast strains have either lost large parts of, or completely lost their mitochondrial DNA, rendering the yeast incompetent for synthesis of mitochondrial encoded proteins. The yeast, *Kluyveromyces* lactis, **is a facultative anaerobe like *Saccharomyces cerevisiae*, but differs in that it requires mitochondrial DNA even when grown on a fermentable carbon source. Based on this phenotype, the cells are referred to as being “petite negative”. This is in contrast to *S. cerevisiae*, which is petite positive as deletions in the mitochondrial DNA are not lethal. Mutations in *K. lactis* were identified that convert the cells from petite negative to petite positive and the complementation group was named mitochondrial genome integrity (*mgi*) 113,114,115,116,117,118. Surprisingly, the *mgi* mutations mapped in the genes encoding α-, β-, and γ-subunits of the mitochondrial ATP synthase. The *yme*1 mutation converts *S. cerevisiae* from petite positive to petite negative. A number of mutations were isolated from a *yme1* *S. cerevisiae* strain that converted the yeast from petite negative, back to petite positive 119,120. These mutations also mapped to the genes encoding the ATP synthase with some of them being identical to *mgi* mutations identified in *K. lactis*. In a manner analogous to *K. lactis*, the survival of the blood stream form of *Trypanosoma brucei *requires mitochondrial gene expression despite the absence of oxidative phosphorylation during this stage of the life cycle. However, a single polymorphism in the gene encoding the γ-subunit, which mapped in the same region as the *mgi* mutations in the gene encoding the γ-subunit, was identified that allowed for dyskinetoplasty (lack of mitochondrial DNA) in *T. brucei*. 121,122. These diverse systems converging on common mutations, suggest a shared mechanism linking the ATP synthase with loss or retention of mitochondrial DNA.

**Figure 3:**
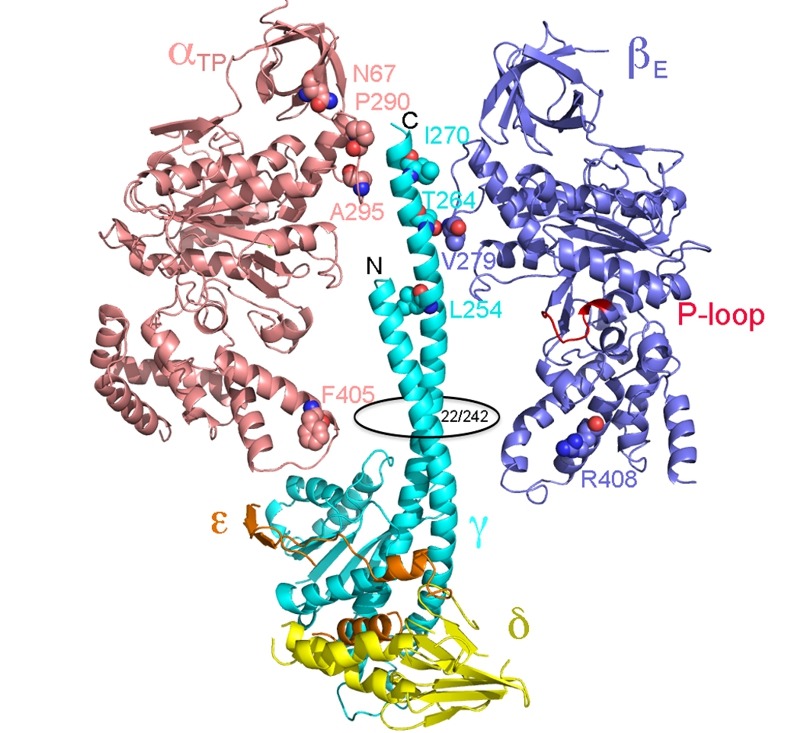
FIGURE 3: Location of residues in the yeast ATP synthase classified as* mgi*. The structural representation is derived from the x-ray structure of the yeast F_1_ ATPase (2HDL). Shown are the αβγδε subunits, as indicated. The α_3_β_3_ core has been stripped down to a single α and β subunit to simplify the view. The *mgi* residues are as labeled. The N- and C- terminal ends of the γ-subunit are indicated as is the region of the orifice of the α_3_β_3_ assembly, which is located at residues 22 and 242 of the yeast γ-subunit. The nucleotide binding P-loop domain is colored red and labeled.

Mutations analogous to *mgi* mutations were made in yeast *S. cerevisiae* and the biochemistry and structure of the enzyme was studied 123. *S. cerevisiae* is petite positive and when *mgi* mutations are introduced into this yeast there is a striking increase in the percentage of petite strains during normal division. A variety of biochemical studies on the isolated mitochondria indicated that the *mgi* mutations caused an uncoupling of the ATP synthase presumably caused by proton leakage through F_o_. Mapping the mutations on the structure of the yeast F_1_ ATPase indicated that the mutations clustered in two regions of the F_1 _ATPase - along the collar region, which steadies the rotation of the central stalk and in the region first identified in bovine F_1_ ATPase and referred to as Catch 2 region 12 in the β-subunit which interacts with the γ-subunit (Figure 3) 123. X-ray crystallography of some of the mutant structures provided a structural basis for the mechanism of uncoupling rotation of the rotor from ATP hydrolysis or synthesis 124. Interesting, two of the *mgi* mutations (α-Asn67Ile and β-Val279Phe) appear to disrupt phosphate binding to the E site which, if correct, would suggest that these mutations allow for proton driven rotation in the absence of substrate (phosphate) being bound thereby resulting in a less efficient, less coupled, enzyme.

While the structural and biochemical studies gave an insight into the effect of *mgi* mutations on the biochemistry of the ATP synthase, it is still not clear how the mutations convert a petite negative strain into a petite positive strain. There is a correlation between the percentage of petite mutations formed and the severity of the mutation on the coupling of the ATP synthase, but this is distinct from how uncoupling allows the petite negative cells to lose their mitochondrial DNA.

### Mutations in the γ-subunit 

The coiled-coil motif formed by the N- and C-terminal helices of the γ-subunit, rotates within the α_3_β_3_ complex. The rotation is believed to proceed with minimal resistance but maintains critical interactions to cause catalysis. Tethering the γ-subunit to the residues on either the α- or β-subunits inhibits rotation and blocks catalysis. When disulfide bridges were introduced in the *E. coli* ATPase between γ-Leu262Cys and α-Ala334Cys or between γ-Cys87 to β-Asp380Cys, the ATPase driven rotation was nearly completely inhibited 125. However, rotation was not blocked when a covalent cross-link was made between γ-Ala285Cys and α-Pro280Cys. The location of the cross-links apparently provides an explanation to resolve this paradox. The cross links to γ-Leu262Cys and γ-Cys87 were positioned in the center or below the center of the rotor while the cross-link to γ-Ala285Cys was positioned at the top of the rotor. This study suggested that the amino acid residues at the top of the rotor could swivel along the c-α bonds or around the side chain bonds 126,127.

There are two isoforms of the γ-subunit in humans and other mammals: a “heart isoform” and a “liver isoform”. The isoforms differ by a single amino acid; the liver isoform has an additional residue, an aspartate, at the C-terminus. The isoforms are formed by alternative splicing, which is conserved and highly regulated 128,129,130,131. The heart isoform is expressed in heart, skeletal muscle, and intercostal muscle, diaphragm, all containing tissues with a high and variable energy demand. The liver isoform is expressed in brain, thyroid, spleen, pancreas, kidney, testis and liver. Of these, all but the brain consume relatively low amount of ATP and have a steady energy demand 132. The expectation is that these isoforms have biochemical significance and are important for the physiology of the tissues.

Based on the crystal structures of the bovine and yeast F_1_, we can predict that the Asp at the C-terminus of the γ-subunit in the liver isoform forms a salt linkage with α-Arg288. However, comparison of the ATPase activity of the two isoforms by both bulk ATPase activity measurements and analysis of rotation of single molecules did not demonstrate any significant difference in kinetics of ATPase reaction cycle 35,133. This suggests that if the isoforms serve as a regulatory mechanism, that the regulation must occur in conjunction with another molecule.

Earlier studies on the bacterial enzyme have been critical in understanding the structure-function relationship of the γ-subunit of the ATP synthase. The “axle” of the bacterial and mitochondrial enzymes extends for about 22 residues on the N-terminus and for about 43 residues on the C-terminus (Figure 3). The helices formed by these regions form a coiled-coil that spins within the center of the α_3_β_3_ assembly. An early mutation isolated in *E. coli* γ-subunit gene, NR70, resulted in a 7 amino acid deletion, residues 22-28, in the bacterial γ-subunit 134,135. The resultant bacteria were lacking membrane bound F_1_F_o_ and exhibited a reduced ability to accumulate sugars and amino acids but this could be restored by the addition DCCD, a covalent inhibitor of F_o_. 134,135. DCCD is apparently acting to prevent proton leakage through F_o_. As such, this mutation exhibited qualities that are predicted for mutations that uncouple the ATP synthase, which allow free movement of protons.

A number of other studies have more closely defined the structure function relationship of the bacterial γ-subunit. Despite deletion of the axle region of the γ-subunit (the coiled-coil helices) the enzyme was still able to rotate in the correct direction with ATP hydrolysis. However, the rotation rate was greatly reduced and mutants with large deletions exhibited irregular motion 136. Another study demonstrated that major deletions in the axle impaired ATP synthesis only by about 75% 137. Based on the results from these and other studies, it was concluded that the C-terminal region of the γ-subunit is responsible for about half of the torque generated by ATP hydrolysis and the difference attributable to the interactions between the γ-subunit and residues at the orifice of the α_3_β_3_ subcomplex 138,139,140. These results are also consistent with the location of the *mgi* mutations, which map at the top of the area surrounding the axle and at the general area of the orifice of the α_3_β_3_ subcomplex (Figure 3).

## MUTATIONS IN THE ATP SYNTHASE THAT CAUSE HUMAN DISEASE 

### Disease-causing mutations in the nuclear DNA

There have been three reports of mutations in nuclear genes encoding subunits of the ATP synthase causing human disease: that encoding the ε-subunit 141 and more recently, the α-subunit 142,143. In the first case, the mutation Tyr11 of the mature ε-subunit (Tyr12 in the precursor) was mutated to a Cys. Tyr11 is highly conserved in the ε-subunit in which the primary sequence is otherwise poorly conserved. The ε-subunit forms a two-helix hairpin and the N-terminus interacts with both the γ- and δ-subunits of the F_1_ ATPase. The side-chain phenolic group of Tyr11 is in the center of a pocket formed by residues (based on the structure of the bovine F_1_ ATPase, pdb: 1E79 17) Trp4, Ser10, Ile12, Arg13, Tyr14, and Ser15 of the ε-subunit, Val57, Leu58, Ser79 and Gly80 of the δ-subunit, and Asn203 and Glu206 of the γ-subunit. The mutation, ε-Tyr11Cys, resulted in a low level of the intact ATP synthase in the fibroblast cells of the patient, but there was an apparent associated increase in the level and activity of Complex III and IV of the electron transport chain. The ATP synthase that was assembled had reduced amount of associated ε-subunit, but nonetheless, was apparently functional. The patient was a 22 year old that presented with symptoms typical of a patient with a mitochondrial disease. This disease is not lethal and thus consistent with the impairment, but not elimination, of activity of the ATP synthase. The mutation likely alters the assembly of the intact ATP synthase.

The second example of a nuclear mutation in the ATP synthase causing a human disease was in the α-subunit, Tyr278Cys (Tyr321Cys of the pre-protein) 142. In this case, the patient had symptoms that were typical of a severe mitochondrial disease resulting in death of the child at age 3. The patient had one sister with similar symptoms who died at 15 months. Both patients paradoxically had combined respiratory chain deficiency. Despite more severe symptoms and outcome, the Tyr278Cys mutation had a much less apparent effect on the function of the ATP synthase. The most pronounced effect of the mutations was observed in both the muscle and liver of the patient, where there was a moderate depletion of the mitochondrial DNA. A similar finding was observed when the comparable mutation was modeled in yeast 142. Tyr278 is within the region where the *mgi* mutations were clustered in the yeast F_1_ ATPase suggesting that the mutation might affect the coupling capacity. The increased loss of mitochondrial DNA is also consistent with the *mgi* phenotype and thereby suggesting that the mutation might decrease the coupling of the ATP synthase. However, while there was some indication that the corresponding mutation when made in yeast uncoupled the mitochondrial ATP synthase, the effect was not large. One possible explanation for this paradox is that even mild uncoupling of the ATP synthase causes loss of mitochondrial DNA over many generations, acting analogous to a degenerative disease, and the loss in the mitochondrial DNA is ultimately the reason for the disease symptoms.

The third example was a disease presented in two siblings who contained a mutation in the α-subunit, Arg286Cys (Arg329 in the pre-protein) 143. Both children died in the first weeks of life due to severe encephalopathy with associated extensive cerebral damage. In addition, there was damage to the lungs, kidney, and skeletal muscle. The mutation caused a dramatic decrease in the cellular level of the ATP synthase as measured by activity. The mutation was inherited from the father who was a heterozygote carrier. The mother did not have the effecting mutation but apparently had another mutation that caused loss of expression of the gene encoding the α-subunit from one of her homologs. Consistent with this, the level of mRNA for the α-subunit was decreased by 40% in the blood cells from the mother as compared to the father. As such, both patients were heterozygous for the Arg286Cys causing mutation and did not express any wild type form of the α-subunit. Complementation studies using cells derived from the patients indicated that the mutation in the gene encoding the α-subunit was responsible for the cellular defects. Unlike the mutation discussed earlier, this mutation did not have any apparent pleiotropic effect on the level of mitochondrial DNA or complexes of the electron transport chain.

These two reported disease-causing mutations in the gene encoding the α-subunit were recessive. Because there are three copies of the α-subunit in F_1_, assuming equal assembly between the mutant and wild type forms of the α-subunit, just 1/8th of the F_1_ would be assembled with all wild type α-subunit. As discussed earlier, a single mutation that inactivates the catalytic reaction will completely inhibit the enzyme activity 47. As such, assuming equal probability of assembly of the wild type with the mutant forms, catalytic site mutations will inhibit activity by 7/8 of the total. Apparently, at least for these mutations, incorporation of a mutant copy of the α-subunit into the ATP synthase complex did not inactivate the catalytic site. Also, since the mother in the family with the Arg286Cys disease causing mutation expressed just 60% of the level of the α-subunit, a null mutation in the gene encoding the α-subunit causing a 50% decrease is unlikely to be a problem due to haploinsufficiency. Furthermore, there have been no reports to date on dominant mutations in the ATP synthase that are responsible for disease.

### Disease-causing mutations in the mitochondrial DNA

The mitochondrial genome of humans encodes 2 subunits of the mitochondrial ATP synthase - subunits a and 8, with the remaining being encoded in the nucleus (c.f. **Table 1**). The majority of mutations in the ATP synthase that are associated with disease is located in the mitochondrial ATP6 gene, which encodes subunit-a. This is likely reflective of the importance of subunit-a in the proton translocation.

Disease-causing mutations in the genes encoded in the mitochondrial DNA were first identified in 1990 144. The mitochondrial genome is very compact, such that the open reading frames for subunits -a and -8 of the ATP synthase overlap with each other. To date, 29 disease- or phenotype-causing mutations have been reported in the ORF for subunit-a, subunit 6, and in the region that encodes both subunit-a and -8 (see www.mitomap.org for current listing).

**Figure 4:**
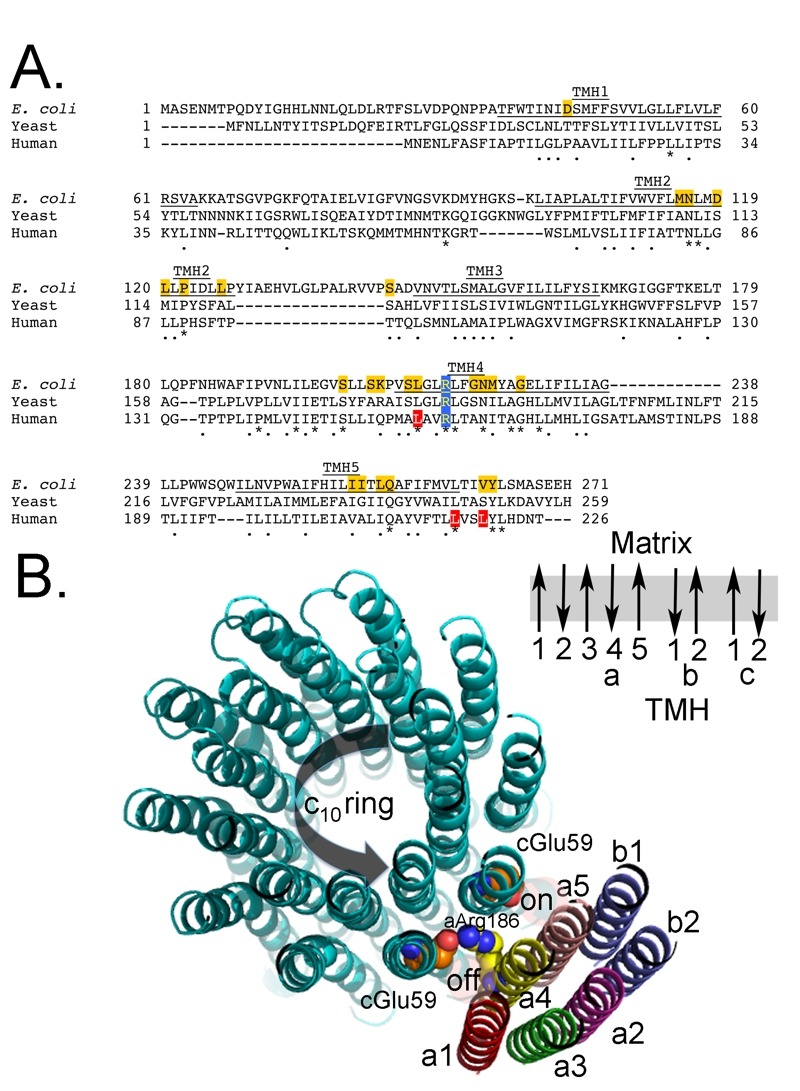
FIGURE 4: Primary sequence alignment of subunit-a. **(A)**The partial primary sequence alignment of subunit-a from *E. coli*, yeast, and human is shown. The residues predicted to form transmembrane helices 3-5 (TMH3-5) are underlined and labeled 54,56,63,145. The highly conserved and essential R210 (*E. coli* numbering) is shaded blue. The residues shaded red are mutated in the discussed human diseases. The residues shaded gold are identified as important for proton movement 54,204. **(B)**(B) Hypothetical model of the arrangement of the helices from subunits a, b, and c. The helices from subunits a (5 helices), b (2 helices) and c (20 helices) are shown along with the side chains for a-Arg1876 (yellow/blue) and c-Glu59 (red). There is very little cross-linking data positioning Helix 1 of subunit-a and thus the position is inferred from the absence, rather than presence, of cross-linking. Subunit b is thought to have a stoichiometry of 2 in the *E. coli* enzyme and 1 in the mitochondrial enzyme and there is 1 transmembrane helix in the bacterial subunit and 2 in the mitochondrial subunit. This view is from the matrix side of the mitochondrion. Also shown is the relative direction for deportation/protonation of c-Glu59 during ATP synthesis. The black arrow indicates the direction of rotation during ATP synthesis. Also shown is a schematic of the orientation of the transmembrane helices (TMH) for subunits a, b, and c in the membrane. Note that the helices for the subunits-a and -b are representations of a possible placement and secondary structure, while the structure of the c-ring is a crystal structure obtained from yeast 8.

Figure 4a shows a comparison of the primary sequence of subunit-a from *E. coli*, yeast, and human. Subunit-a is thought to be comprised of 5 transmembrane helices (TMH1-5) and the predicted TMH3-5 are as indicated. Figure 4b shows the possible arrangement of the transmembrane helices of subunits a (5 helices), b (2 helices) and c (20 helices) as determined by cross-linking and other biochemical studies 54,145,146,147,148,149. The structure of subunit-a is not known, so this is just a hypothetical representation of the location of the putative helices. a-Arg186 in yeast (210 in *E. coli*, shaded blue) is conserved and thought to participate directly in the mechanism of proton pumping and thought to be closely associated with Glu59 in subunit-c. As such, in the model, the side chain of a-Arg186 is shown close to the carboxylate of c-Glu58. The residues shaded in yellow have been implicated to be associated with the proton half-channel (shown in the model as “On” and “Off” for the direction of proton movement during ATP synthesis) based on chemical modification studies using the enzyme isolated from *E. coli* 54,150. Mutations in 8 residues in the human subunit, including a-Leu156, a-Leu217, and a-Leu220, are associated with diseases 144,151,152,153,154,155,156,157,158,159,160,161,162,163,164,165,166,167,168,169,170,171,172,173,174,175,176,177,178,179,180,181,182,183,184,185,186,187,188,189. Residues a-Leu156 and a-Leu217 are highly conserved, adjacent to highly conserved, and located in TMH4 and TMH5 which contain clusters of residues that have been implicated in proton movement. a-Leu220 is adjacent to TMH5 and abuts highly conserved residues a-Tyr221 and a-Leu222. Based on the clustering of critical residues and the disease forming mutations, it seems likely that TMH4 and TMH5 are principle players in forming the proton half channels. An attractive hypothesis is that mutations in these residues alter proton movement indirectly by altering the secondary structure, for example a-Leu156Pro, a-Leu217Pro, and a-Leu222Pro or directly by impeding proton movement, as with a-Leu156Arg or a-Leu217Arg.

The diseases associated with mutations in the gene encoding subunit-a vary in severity depending upon the allele, but the percentage of the mutant copy in each cell can vary greatly for 2 reasons. Mitochondrial DNA is typically a heteroplasmic mixture of mutant and wild type copies and further, mitochondrial DNA does not segregate in a Mendelian manner. For example, the mutations Leu156Arg, Leu156Pro, Leu217Arg, and Leu217Pro, manifest as NARP (Neurogenic muscle weakness, Ataxia, and Retinitis Pigmentosa) at low heteroplasmy but as Leigh syndrome (severe and fatal encephalopathy) when the percentage of mutant copies of mitochondrial DNA exceeds 90% (for review, see 190,191,192). The disease phenotype is confounded by variations in the percentage of mitochondrial DNA molecules in the cells that contain the mutation, which can vary considerably between tissues and even between cells within a tissue - a situation referred to as “mosaic”. The varying genetic background of the patients, the tissue mosaicism, and the resulting variations in heteroplasmy can make interpretation of biochemical data and diagnosis difficult. To circumvent these problems, the effect of the mutation on the function of the ATP synthase has been evaluated by making the corresponding mutation in yeast or *E. coli* followed by biochemical analysis of the resulting mutant enzyme.

Using yeast as a model system, the effect of a deletion mutation in the gene encoding subunit-a on the assembly and function of the ATP synthase was assessed 193. Subunit-a was shown to be necessary for the activity, but not for the assembly of the ATP synthase, and subcomplexes of the enzyme can be formed devoid of subunit-a. The ATPase activity was at near normal levels but it was nearly insensitive to the F_o_ inhibitor, oligomycin. The cells rapidly lost mitochondrial DNA when the selection for the mitochondrial DNA was relaxed. The cells lost cytochrome oxidase activity, as was noted for deletion mutations in genes encoding other subunits of the ATP synthase discussed earlier. However, this study demonstrated that the loss of cytochrome oxidase activity was due to the loss in the synthesis of mitochondrial encoded subunit 1 of cytochrome oxidase, Cox1p. This causal relationship was further supported by studies in yeast and human fibroblast cells that showed that cytochrome oxidase activity was decreased in cells treated with oligomycin and partial recovery was caused by addition of an uncoupler 194. In yeast, oligomycin caused a loss of Cox1p synthesis similar to that observed with the prior study with subunit-a deficient cells. These studies suggest that hyperpolarization of the mitochondrial membrane may compromise the synthesis of subunit 1 of cytochrome oxidase as addition of a limiting amount of uncoupler can restore cytochrome oxidase. Hyperpolarization of the mitochondrial membrane can be the result of mutation that inactivate or inhibit activity of the ATP synthase, thus establishing a possible link with cytochrome oxidase. Thus, as discussed earlier, there appears to be an evolutionary conserved link between a functional ATP synthase and synthesis of cytochrome oxidase and possibly the ΔΨ of the mitochondrial membrane is that link.

The biochemical consequences of a-Leu156Arg mutation were studied by making the corresponding mutation in *E. coli* 195. The mutant enzyme had enzymatic properties that were nearly identical to the enzyme devoid of subunit-a. As such, in *E. coli*, mutations that affect the folding of subunit-a and assembly of the holo-enzyme cannot be firmly distinguished from mutations in subunit-a that do not prevent assembly, but rather impair the activity of the enzyme, simply by measuring the levels of ATP hydrolysis and synthesis. The analysis of the cultured fibroblasts 177 and cybrids 196 containing the Leu156Arg mutation indicated that the P/O ratio of the affected mitochondria 177 was not altered, but there was a decrease in both the level of ATP synthase and respiratory activity, and the mutation seemed to destabilize the complex 196. Interestingly, the Leu156Arg mutation made the ATPase activity hypersensitive to oligomycin 177,196. One explanation for this effect that is consistent with the mode of oligomycin binding to subunit c 52 is that the mutation in subunit-a made the binding site more accessible to oligomycin.

Similarly, subunit-a mutations, Leu217Arg, Leu217Pro, Leu156Arg, and Leu156Pro, were also studied using *E. coli* enzyme 161. The mutation Leu217Arg did not largely alter the level of the ATP synthase, but severely inhibited ATP hydrolysis and ATP synthesis by the membrane bound enzyme. These results may indicate that the mutation blocks the proton conduction pathway in the ATP synthase without uncoupling the enzyme. In contrast, the Leu217Pro mutant form had considerable ATP synthase activity thus clearly showing biochemical differences due to the allelic differences. The mutations Leu156Arg and Leu156Pro caused diminished ATPase and ATP synthase activity but had little effect on the coupling of the enzyme. When the corresponding mutations, Leu156Pro and Leu156Arg, were made in yeast *S. cerevisiae* and analyzed, they demonstrated a decrease in the level of ATPase and ATP synthase activity, and again, a pleiotropic effect on the level of cytochrome oxidase activity 197,198. For the yeast enzyme with Leu156Arg, the enzyme appeared to be fully assembled but defective in proton pumping and ATP synthesis. For the yeast enzyme with Leu156Pro, the decreased level of ATP synthase activity paralleled the decrease in oligomycin sensitivity suggesting that subunit-a was not efficiently incorporated into the ATP synthase. The effect of these mutations on the biochemistry of the bacterial and yeast enzymes were entirely consistent with the proposed role of subunit-a as providing a half-channel for proton access to the essential carboxylate in subunit-c, access to the intermembrane space, and the matrix space of the mitochondrion.

Disease-causing mutations in subunit 8 are much less common than those in subunit-a. The mutation Trp55X, where X is a stop codon, results in the truncation of 14 amino acids from the C-terminus of subunit 8 199. The resulting effect is a destabilization of the ATP synthase complex such that there was a large decrease in the level of intact ATP synthase complex and an increase in the amount of free F_1_ ATPase, as judged by Blue Native gels. It would be rather remarkable if the Blue Native gels accurately reflected the level of free F_1_ ATPase in the cells as a high level of free F_1_ would lead to a high level of futile and wasteful ATP hydrolysis. Possibly the ATP synthase complex is intact in the cell and only falls apart during the gel analysis. Alternatively, it is possible that the natural inhibitor protein, Inh1, is able to prevent the hydrolysis of ATP by free F_1_ ATPase. No other biochemical studies were done to give any insight into the effect of this mutation on the function of the ATP synthase.

There was a mutation in the overlapping region of the open reading frames of subunit-a and subunit-8 200. The mutation created a Thr55Arg replacement in subunit-8 and a Met1Thr replacement in subunit-a. The fibroblasts from the patient showed a significant, but not dramatic decrease in the ATP synthase activity driven by malate/glutamate or succinate. Likely, the change in the initiating codon for subunit-a resulted in a decrease in the level of subunit-a and thus in a decrease in the intact ATP synthase complex.

### Single nucleotide polymorphisms (SNPs) in the nuclear genes encoding subunits of the ATP synthase

With the advent of rapid sequence analysis of the human genome, the bottleneck for the understanding of individual genetic differences is now the analysis of the effect of single nucleotide polymorphisms on biochemistry of the enzyme and the resulting effect on the physiology of the cell and organism. This analysis will be greatly aided by the understanding of the structure/function relationship of the protein and by the library of mutations that have already been studied. Nonetheless, the analysis is difficult and generally unpredictable - as was the case for the disease-causing mutation identified in the gene encoding the α-subunit and discussed earlier 142. The difficulty can be illustrated by looking at the current library of the SNPs in the gene encoding the human γ-subunit of the ATP synthase (ATP5C1).

**Figure 5:**
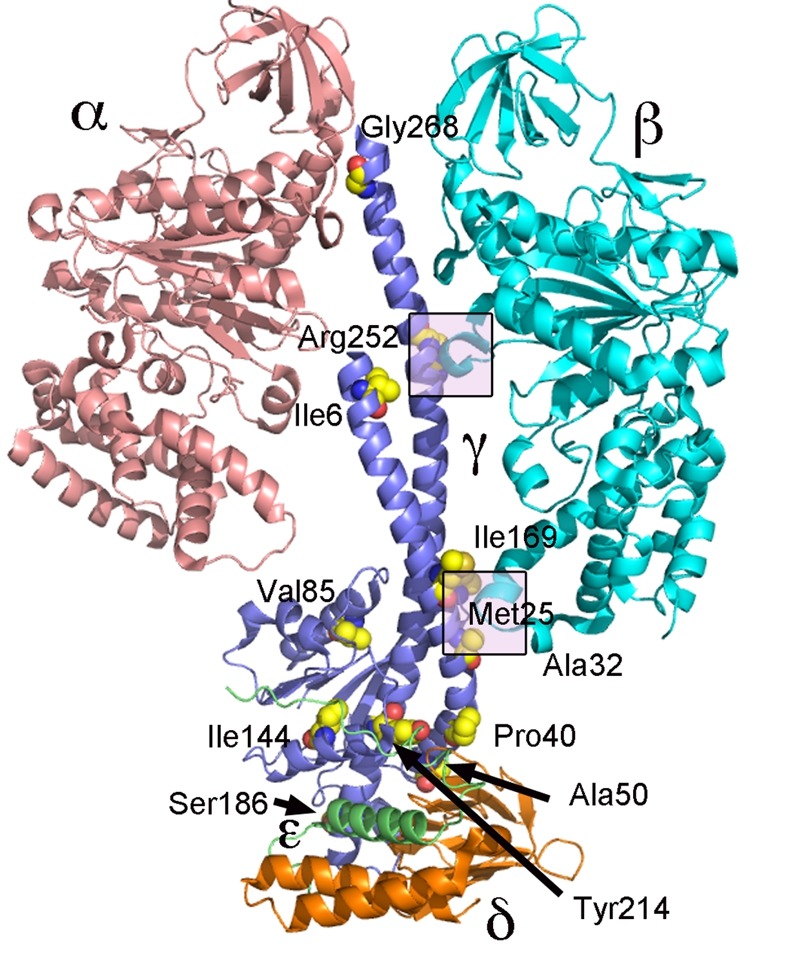
FIGURE 5: Relative location in the ATP synthase of residues mutated by known single nucleotide polymorphisms. The structural representation is derived from the x-ray crystal structure of the bovine ATPase (1E79). To simplify the image, only a single copy of the α- (salmon) and β- (cyan) subunits is shown along with the γ- (purple), δ- (orange), and ε- (green) subunits. The residues, which are altered by known SNPs in the γ-subunit are shown in sphere representation and numbered using the human numbering system with 1 as the first residue in the mature peptide (the initiating Met is -25 for the γ-subunit). The two boxes represent the regions corresponding to Catch 1 and Catch 2 regions.

Many SNPs have been mapped in ATP5C1 (see http://evs.gs.washington.edu/EVS). Note, mutations in genetic databases are normally numbered using the initiating Met as 1, while the convention used here and in structural and biochemical studies is that the first amino acid in the mature peptide is numbered 1. In the case of the human γ-subunit, there is a 25 amino acid leader polypeptide that targets the subunit to the mitochondria. Current SNPs that create missense mutations in the mature γ-subunit are: Ile6Val, Met25Thr, Ala32Val, Pro40Ser, Ala50Val, Ile85Val, Ile144Val, Ile169The, Ser186Asn, Tyr214Cys, Arg252Cys, Gly268Ser (Figure 5). γ-Met25 interacts with β-Ile390, which is in the Catch 2 region. The mutation in *E. coli* γ-subunit that corresponds to Met25Lys partially uncoupled the enzyme, reduced the binding affinity for Pi and reduced the apparent affinity for ATP of the *E. coli* enzyme 201,202,203. However, it is not known if Met25Thr affects the biochemistry of the human enzyme.

The SNPs are not limited to the γ-subunit, but occur in some of the other genes encoding other subunits of the ATP synthase including the α- and β- subunits. Notwithstanding the putative importance of any one residue in the ATP synthase, the effect of a residue-altering mutation on the biochemistry of the enzyme is uncertain. Furthermore, the relationship between function of the ATP synthase and physiology of the organism is unclear. How much change in the coupling or activity of the ATP synthase is needed to have an observable effect on the individual? Do these SNPs account for differences in the individual’s performances in different climates, physical activities, or recovery from a trauma, such as a heart attack? The future lies in the ability to link SNPs with physical and medical risks or advantages for any one individual. Can we use yeast as a model organism to assess the effect of SNPs on the structure and activity of the enzyme?

A major impediment to the understanding of the ATP synthase is the lack of a high-resolution structure of the entire enzyme complex. The most pertinent portion of the enzyme that is lacking structural details is the proton half-channels, and this severely limits our understanding of the mechanism of proton translocation and pumping. The recent advances in electron microscopy make it inevitable that the structure of the ATP synthase will be solved but there is still hope that a high-resolution crystal structure will be obtained. However, structure determination of the mutant forms of the enzyme will likely also be necessary to fully understand the effect of disease causing mutations, especially in subunit a, and the effect of SNPs on the structure and function of the enzyme.
